# CN+: Vehicular Dataset at Traffic Light Regulated Intersection in Bremen, Germany

**DOI:** 10.1038/s41597-024-03498-4

**Published:** 2024-06-22

**Authors:** Thenuka Karunathilake, Meyo Zongo, Dinithi Amarawardana, Anna Förster

**Affiliations:** 1 University of Bremen, Sustainable Communication Networks, Bremen, 28359 Germany; 2 University of Ngaoundere, Mathematics and Computer Science, Ngaoundere, 454 Cameroon; 3 University of Kassel, Department of Hydrogeology, Kassel, 34125 Germany

**Keywords:** Research data, Scientific community

## Abstract

Vehicular Ad-Hoc Networks (VANETs) were introduced to avoid vehicular-related accidents and to improve the safety of both vehicular passengers and other road users. In VANETs, the vehicles are expected to communicate with neighbouring vehicles to increase awareness about the surrounding by using V2V (vehicle-to-vehicle) communication links. Since the introduction of VANETs, much research has focused on developing state-of-the-art algorithms to increase safety. However, real-world testing of these developed algorithms has become challenging due to the required high cost and multiple practical reasons. Therefore, simulation-based testing is commonly used for VANETs related applications. Using real datasets inside a simulation can significantly increase the results’ accuracy and help to achieve realistic results. In this study, we present a dataset called ’CN+’, which consists of more than 25,000 vehicles collected over 32 hours at a signalized intersection in Bremen, Germany. paper

## Background & Summary

The increasing number of vehicles worldwide has recently become a significant safety concern for both vehicular passengers and other road users (pedestrians, cyclists, etc.) due to the increased number of fatal accidents^1^. Therefore, many systems were introduced during the past decades to enhance vehicular safety. Most of these efforts focused on introducing state-of-the-art Advanced Driver Assistance Systems (ADAS) by deploying multiple sensors (radars, lidars, and cameras) in the vehicles. With the help of in-built sensors, these ADAS approaches increase the driver’s awareness about the surrounding environment, aiding the driver in making safe driving decisions and avoiding collisions. However, in complex driving environments (intersections, highways, etc.) and challenging weather conditions, fatal crashes still happen even with the most sophisticated ADAS due to the many practical limitations of deployed sensors^2,3^.

The idea of Connected Automated Vehicles (CAVs) was introduced to further increase vehicular safety with the ADAS by enabling communication among vehicles and creating VANETs. The communication links among vehicles (V2V) and between vehicles and infrastructure (V2I) are used to exchange useful information to improve overall vehicular safety by increasing awareness. Since the introduction of VANETs, numerous safety-related CAV applications were proposed and developed by the research community^4,5^. These safety-critical applications must be tested and evaluated extensively before any real-world implementations. The most accurate testing method is real-world testing because it allows the proposed systems to be evaluated in all realistic scenarios. However, real-world testing has several challenges: it requires high costs, privacy concerns, and many other practical reasons. Therefore the research community has focused on simulation-based testing for many VANET applications. As a result, significant effort was focused on developing simulators and frameworks to simulate many aspects of VANET scenarios, including communication protocols and numerous Intelligent Transportation System (ITS) applications^6–9^.

Vehicle mobility has become one of the difficult aspects to model inside a simulator due to the complex driving characteristics of people. For example, areas like airports, train stations, or the city centre might be more congested than other areas and some intersections in the city might be busier during certain times of the day due to rush hours. Even though multiple mobility models are already available on the simulators, modeling these special characteristics is challenging and might not be considered inside the mobility model. Hence, obtaining realistic results is strictly limited due to the unrealistic movement patterns of vehicles inside the simulator. Therefore, many research communities focused on using real datasets for VANET simulations instead of synthetic mobility models because real datasets include realistic driving behaviours, which can lead to more accurate results. However, gathering these real datasets requires significant effort for many practical reasons, including privacy, obtaining permission, and severe weather conditions. Currently, several publicly available datasets exist for VANET scenarios^10–16^ and Table 1 summarises these available datasets. Most available datasets focused on intersections, as shown in Table 1 because simulating intersection scenarios is crucial since most road accidents happen near intersections due to complex and challenging driving conditions. Similarly, the number of vehicles and duration of the dataset are also essential factors when selecting datasets for simulations. The number of vehicles on the dataset limits the number of vehicles accommodated during the simulation run. As shown in Table 1, the available datasets contain vehicles between 350^10^ and 110,500^14^ and in case of intersection scenarios 350^10^ and 18,642^11^.

**Table 1:** Comparison of different available datasets for VANET scenarios.

Title	City and Country	Location	Number of Vehicles	Duration	Locations	Road User Type
Ko-PER^10^	Aschaffenburg, Germany	intersection	350	-	1	pedestrian, bicycle, vehicle
Interaction^11^	United States, China, Germany and Bulgaria	intersection	18 642	8 hours	4	vehicle
DUT^12^	Dalian University of Technology, China,	campus	1 862	-	2	pedestrian, vehicle
Stanford Drone^13^	Stanford University, Stanford, United States	campus	10 240	-	8	pedestrian, bicycle, skateboard, cart, vehicle
highD^14^	Cologne, Germany	highway	110 500	147 hours	6	vehicle
inD^15^	Aachen, Germany	intersection	13 599	10 hours	4	pedestrian, bicycle, vehicle
SIND^16^	Tianjin, China	intersection	13 248	7 hours	1	bicycle, motorcycle, vehicle
CN+^17^	Bremen, Germany	intersection	25 935	32 hours	1	vehicle

Moreover, the duration of the dataset controls the simulation time. Therefore, more extended datasets are preferred because, with longer datasets, the simulations can be run for an extended duration to test the proposed algorithms in demanding conditions (cache drops, interference, etc.). However, as discussed, collecting datasets is challenging, and at the moment, the most extended dataset available is 147 hours^14^ for highway scenarios and 10 hours^15^ for intersection scenarios.

To further extend the available VANET datasets and to improve the shortcomings of current datasets (number of nodes and duration), we decided to collect and publish the CN+ dataset^17^ containing 25,935 vehicles recorded over 32 hours in a signalised intersection in Bremen, Germany. CN+ is one of the significant intersection-based VANET datasets compared to other available datasets regarding the number of vehicles and duration, as shown in Table 1.

The rest of the paper is arranged as follows. In the Section ‘Methods’, we explain the data collection process in detail. Detailed information about the dataset is described in the Section ‘Data Records’. The Section ‘Technical Validation’ contains information regarding the steps followed to ensure the reliability of collected data and information for researchers is provided in the Section ‘Usage Notes’ on how to use the dataset.

## Methods

**Fig. 1:**
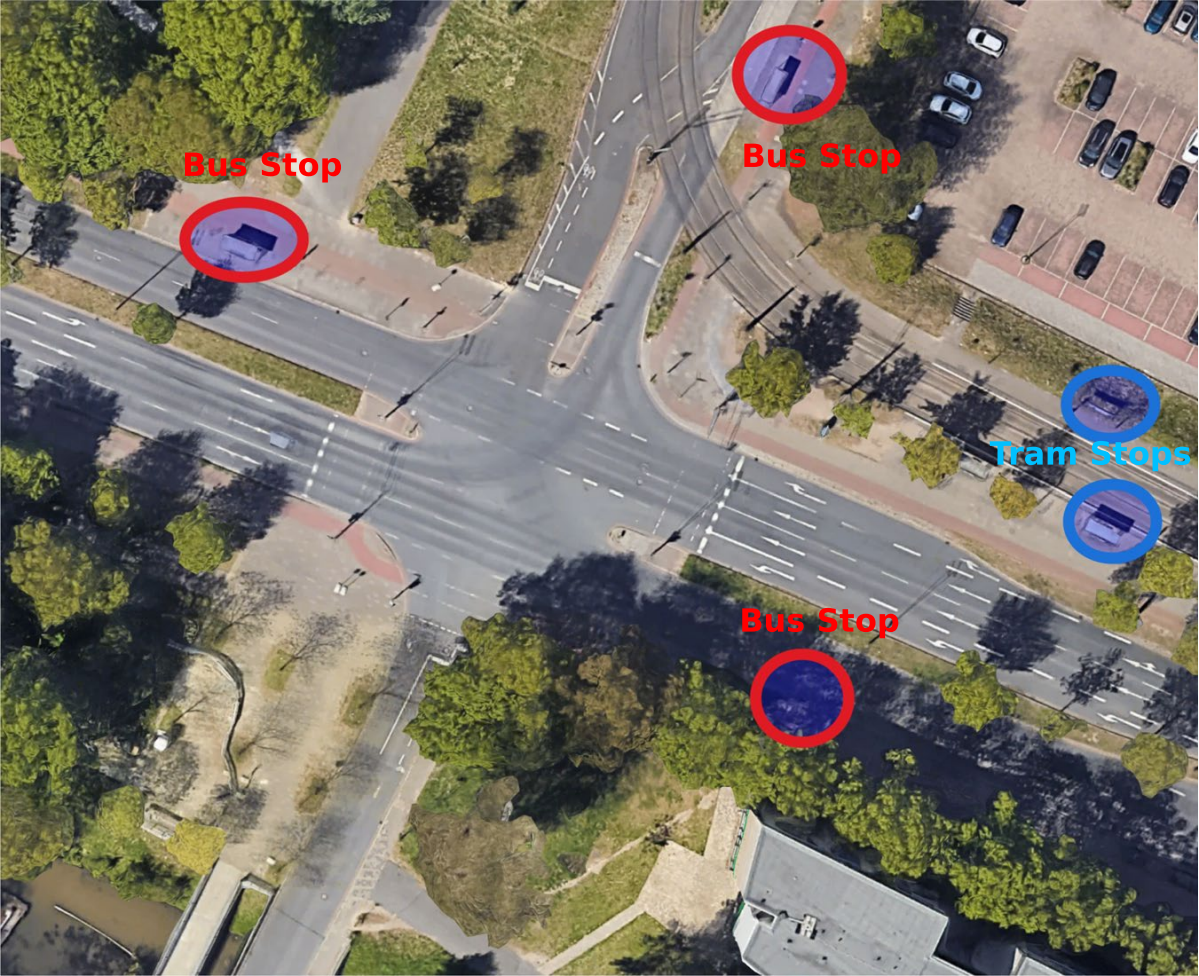
Top view of the selected intersection taken from Google Maps.

As the initial step of the data collection process, we searched and identified several suitable intersections around Bremen, Germany. Among these intersections, we selected one intersection (shown in Fig. 1) for the data collection for several reasons.

- This intersection is a traffic light-regulated 4-way intersection. Two connecting roads join the Bremen city centre and the highway; therefore, many vehicles can be expected on these roads. The other two connecting roads are comparatively low in traffic since these roads connect different University buildings.
- Traffic at this intersection significantly differs between weekdays and weekends because there is very low activity at the University during the weekend. Therefore, the collected dataset contains low-density and high-density traffic scenarios, which can be useful for testing VANET applications in both configurations.
- Multiple public transportation vehicles run through the intersection as shown in Fig. 1. The intersection consists of tram lines with two tram stops (blue circles in Fig. 1) connecting University and the Bremen Airport. Furthermore, multiple bus lines run through the intersection and, therefore, in the surrounding multiple bus stops (red circles in Fig. 1) can be identified.

Our data collection process started on 24.05.2022 and lasted until 06.08.2022. During this period, we collected data in multiple one-hour-long experiments. We only collected data during the morning rush hour periods (08:00-09:00, 09:00-10:00, 10:00-11:00) and in the evening rush hour periods (15:00-16:00, 16:00-17:00, 17:00-18:00, 18:00-19:00). At the end of the data collection period, more than 60 one-hour-long experiments were conducted.

However, after filtering out some of the recorded data, 49 different one-hour-long experiments were selected to include in the CN+ dataset. These 49 slots were selected in a special way, once concatenated dataset adds up to one whole week (Monday to Sunday) of data. As mentioned above, we collected 7 hours of data for each day (3 hours in the morning rush hour and 4 hours in the evening rush hour), and therefore, all together for 7 days, there are 49 one-hour-long experiments. For example, for Monday, the dataset contains 7 hours of data. However, it is important to point out that the data is not continuously collected data on one single Monday, instead collected on different Mondays during the data collection period. Since the CN+ dataset includes a whole week of data, it can be used to test VANET applications in realistic scenarios with varying vehicle densities and numerous vehicle driving behaviours throughout the week. These variations are highly useful characteristics of people’s driving patterns. For example, there may be more vehicles on weekdays than on weekends. Testing VANETs applications with these special characteristics is crucial to optimise proposed applications to perform efficiently.

Furthermore, we subdivide each one-hour-long experiment into 4 smaller sub-experiments, each 10 minutes long. We continuously collected data during these sub-experiments, and between two sub-experiments, there was a time gap of 5 minutes. This time gap was used as a small break for the data-collecting people because it is practically difficult to collect data for one-hour continuously. Therefore, a one-hour-long experiment contains 40 minutes of useful data, and similarly, the whole CN+ dataset collectively contains data for 32 hours.

**Fig. 2:**
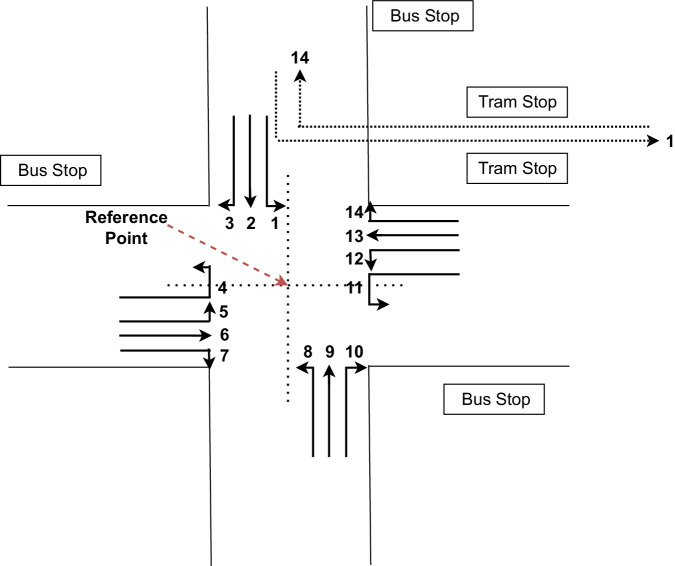
Possible driving directions of the selected intersection.

The selected intersection contains 14 driving directions, as shown in Fig. 2. The two connecting roads which connect Bremen city centre and the highway have 4 possible driving directions (going straight, turning right, turning left, and U-turn). The other two roads, which connect different University buildings, have only 3 different driving directions (U-turns are prohibited). As shown in Fig. 2, we numbered these different directions for easy recognition inside the CN+ dataset. The two tram lines, indicated with dotted lines, are also categorised into driving directions 1 and 14. We used a reference point in the middle of the intersection, as shown in Fig. 2 to manually note down each vehicle’s crossing time of this reference point. For the vehicles not passing through this reference point (vehicles in directions 3, 7, 10, 14, trams, etc.), approximate timing was noted down when the vehicle was in the middle of the corresponding turn. During the data collection, we experienced in some situations due to high-demand multiple vehicles crossing the reference point as a cluster of vehicles. In those situations, since it is difficult to note down individual crossing timings of each vehicle manually, we note down an average crossing timing value for that whole vehicle cluster. It is also important to note that we counted how many vehicles were in each cluster and entered these details into the dataset. Furthermore, we distinguish between public transportation vehicles (buses and trams) and other normal private vehicles during the data collection.

This means all non-public transportation vehicles are categorised into the normal vehicle category including cars, vans, lorries, etc.

We adopted a manual write-down procedure for the data collection mainly to avoid permission and privacy issues caused by other approaches (using cameras and drones), which were used to collect other datasets mentioned in Table 1. We acknowledge that the accuracy of the collected data is relatively low with the manual write-down process compared to recording videos using cameras and drones. However manual write-down process is more beneficial in collecting data for an extended time because weather conditions (rain or fog) and battery issues can limit the data collection process with other approaches, especially in the case of using drones. The CN+ dataset contains data collected during light rain, and collecting data during these weather conditions might not be possible with other approaches.

## Data Records

The complete CN+ dataset includes 25,935 vehicles, and the total duration of the dataset is 32 hours. The CN+ dataset^17^ is publicly available at zenodo.org.

**Fig. 3:**
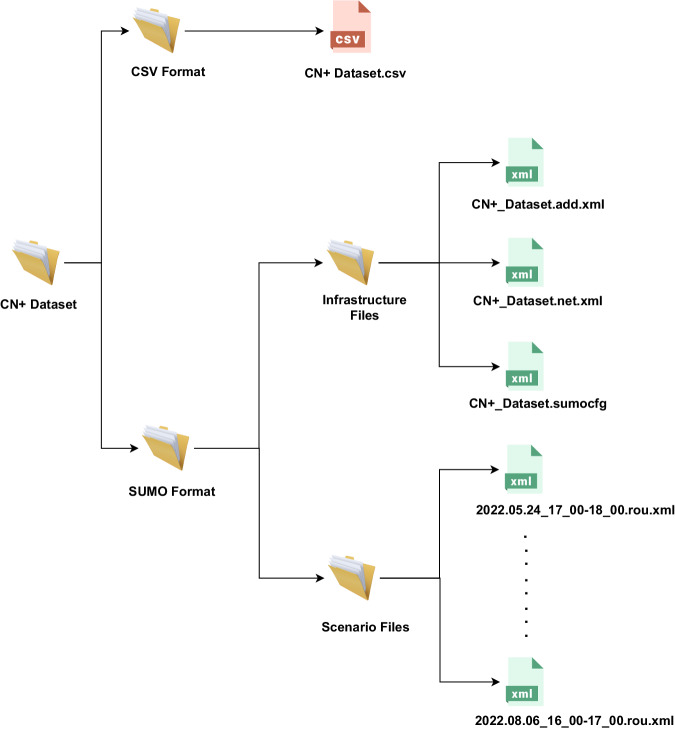
The file structure of the CN+ dataset.

We present the CN+ dataset in 2 different formats to cater for the different needs of the research community. As shown in Fig. 3, the CN+ dataset contains a main folder named ‘CN+ Dataset’. We have included two more sub-folders inside, namely ‘CSV Format’ and ‘SUMO Format’. These folders correspond to the two different formats of the CN+ dataset, and we explain these two formats in detail in the following subsections.

### CSV format

**Fig. 4:**
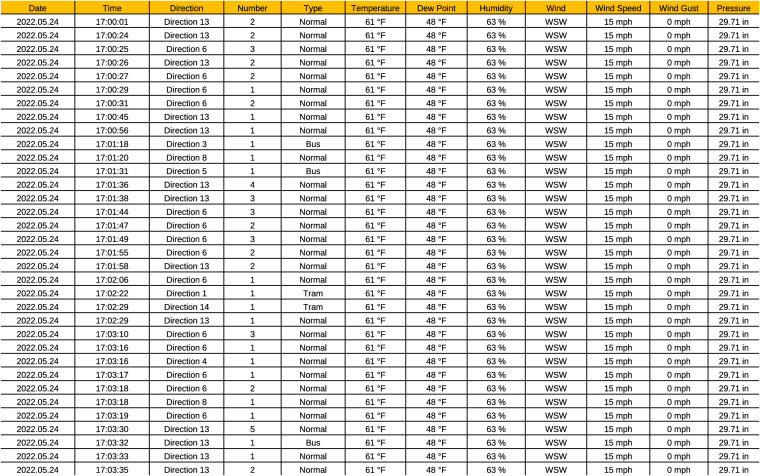
CN+ dataset in CSV format.

In CSV format, we present the complete CN+ dataset in a CSV file as shown in Fig. 4. In this format, the CN+ dataset is arranged in ascending order according to the data collection date. The first column indicates the data collection date, and the second column shows the crossing timings of the reference point (the middle point of the intersection as shown in Fig. 2). The vehicle’s direction is shown in the third column, and there are 14 different directions a vehicle can take, as described earlier. The number of vehicles and the vehicle type are indicated in the fourth and fifth columns, respectively. Our main intention was to include individual crossing timings for each vehicle separately. However, as explained in some situations, we had to cluster several vehicles together because the intersection is traffic light regulated; after the red traffic light, several vehicles might start crossing the intersection simultaneously at the start of the green traffic light. In those situations, we included an average common crossing timing for the cluster of vehicles and indicated the number of vehicles in the cluster in the fourth column, as shown in Fig. 4. In the fifth column, we differentiated between buses, trams, and all other vehicles are categorised as normal. In the case of having different types of vehicles (normal, bus, and tram) inside one cluster, we grouped them according to their type and added them as separate rows in the CSV file with the same common crossing time. Furthermore, we integrated weather information that was retrieved by a weather station located 6 km away from the intersection. This weather information includes temperature, humidity, wind direction, wind speed, and pressure information. Since the weather station does not update regularly, we consider weather data to be the same during a one-hour-long experiment.

### SUMO format

**Fig. 5:**
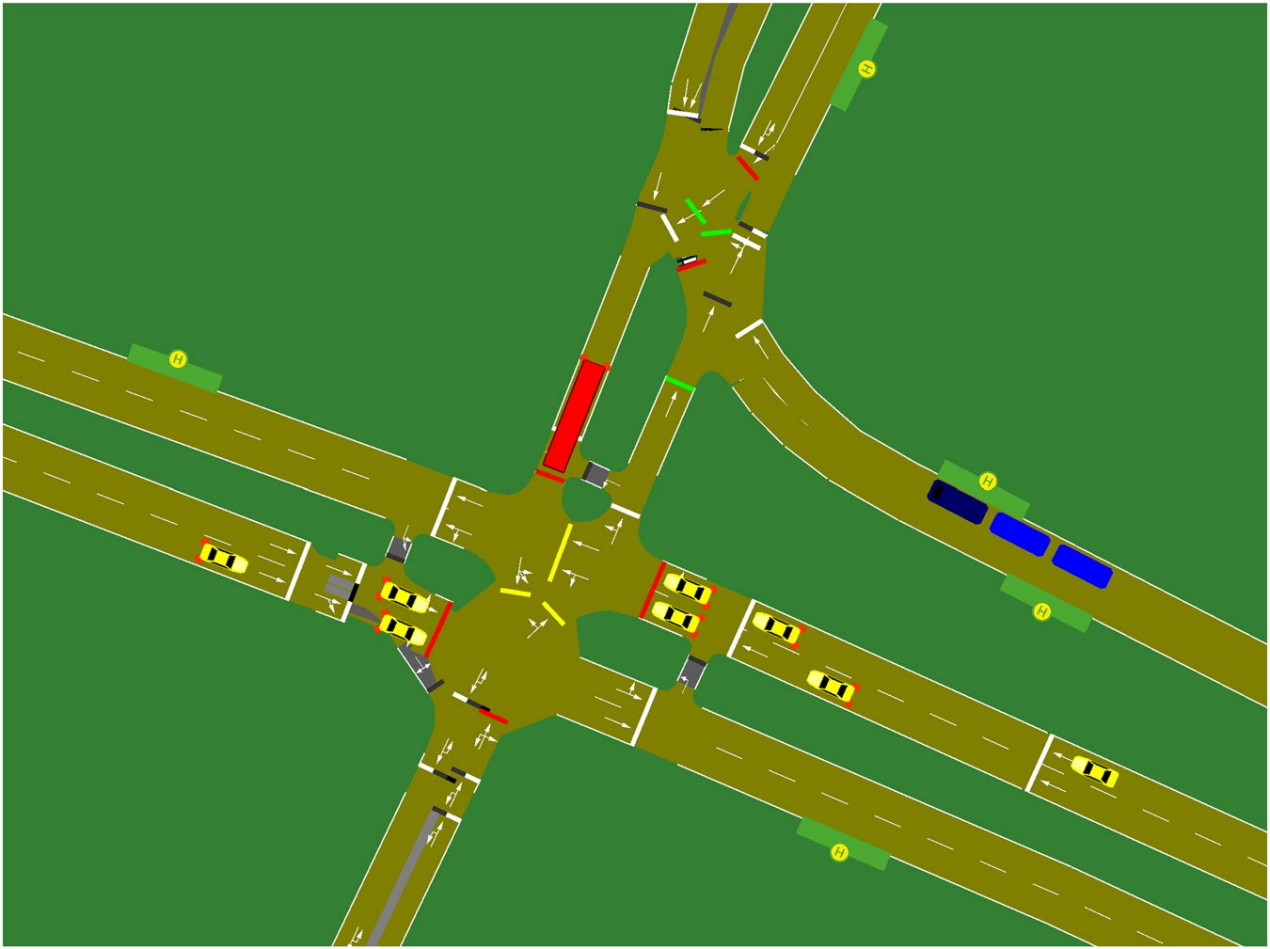
Snapshot of a SUMO converted scenario.

The CN+ dataset is also presented in SUMO^6^ (Simulation of Urban MObility) converted format. SUMO is used to model vehicular mobility, and it can be connected to many network simulators similar to OMNeT++ and NS3. We converted the complete CN+ dataset into SUMO format; therefore, researchers can reuse the dataset directly with simulations. We first import the road infrastructure taken from the Open Street Maps to the SUMO simulator, as shown in Fig. 5. Later all the bus-stops and tram-stops were added manually in their corresponding locations. Furthermore, we adjusted the timing values of traffic lights, average bus-stop, and tram-stop in the SUMO files to approximate real-world values we observed during experiments. Finally, vehicles were added to the SUMO scenarios according to the CN+ dataset values. The crossing timings indicated in the CN+ dataset were used as the departure timings inside the SUMO configurations. This means vehicles including buses and trams were added to the SUMO scenarios using the collected crossing timings. The vehicles are depicted in different colours and icons inside the simulation for easy recognition, as shown in Fig. 5 (bus is indicated with red colour and tram is indicated with blue colour longer icons). Furthermore, the 5 minutes time gaps between two consecutive 10 minutes long sub-experiments were removed during the SUMO conversion process. Therefore, inside the SUMO format vehicles are injected continuously for 40 minutes according to the CN+ dataset values.

## Technical Validation

To collect a reliable and technically accurate dataset, we followed multiple steps, and these steps are categorised into two main parts, Pre-Experiment and Post-Experiment. In the following sub-sections, these steps are discussed in detail.

### Pre-Experiment

The authors initially performed multiple pre-experiments at the same intersection to optimise the final experiment setup, ensuring it was both practical and feasible during manual data collection. During these pre-experiments, the authors collected data for a continuous one hour and identified that collecting data continuously can be a very tiring process, which might lead to collecting unreliable data, especially during peak hours. Therefore, the authors introduced 5-minute gaps between two sub-experiments, as explained in the Method Section. Furthermore, these pre-experiments helped to identify the best location for the data collector to stay during the experiment to see the entire intersection comfortably without any obstruction. In addition, pre-experiments led to the creation of a template paper sheet containing a table to separate different directions, speeding up the manual note-down process. Moreover, with pre-experiments, the authors recognised that collecting reference point crossing time for each vehicle in a group of vehicles is impossible; therefore adopted the procedure to cluster them together and collect one common average crossing time for that specific cluster, as explained in the Method Section. Once the final experiment was designed, we provided comprehensive instructions to all the data collection participants, specifying the precise steps to follow. Altogether, there were 19 data collection participants grouped into 9 different groups. Additionally, a training experiment was conducted at the same intersection with all participants before collecting data for the CN+ dataset. During these training experiments, the authors discussed and clarified all the issues and doubts the data collection participants faced. To further ensure data reliability, the authors accompanied participants during the first actual experiment. Moreover, all the participants were provided with template paper sheets facilitating standarised, efficient, accurate, and fast manual data recording.

These fine-tuning steps with pre-experiments ensured inter-rater reliability during the data collection process. Inter-rater reliability refers to the consistency or agreement between observers when assessing the same dataset. According to^18^, inter-rater reliability is used to measure whether two processes/people and machines identify the same properties in data. In the context of CN+ data collection, inter-rater reliability was ensured by following the same protocols while collecting the data. In our dataset, inter-rater reliability was ensured as follows:

- The authors trained all the data collection participants on how to perform the data collection.
- A training experiment was conducted with all the data collection participants at the intersection.
- All discrepancies and issues that were found during the training experiments were discussed and clarified.

### Post-Experiment

After the data collection, the authors analyse the collected data before including them in the CN+ dataset. Some of the collected data was filtered out during this analysis for various reasons, including unclear handwriting and missing data, as mentioned in the ‘Method’ section. Authors cross-validated each others’ analyses, and only the data that passed the cross-validation was included in the final CN+ dataset. Since some of the data was removed during the analysis and cross-validation process, the authors themselves repeated the data collection process for those missing time slots to complete the missing data to cover the entire week of data. Furthermore, the authors visualized the data once the complete dataset was ready to identify the patterns and characteristics. For example, the number of vehicles at the intersection is higher on weekdays than on weekends, and most vehicles are expected to be in the directions that connect Bremen city center and the highway (Directions 6 and 13), etc. These patterns and characteristics inside the CN+ dataset should match real-life everyday vehicle driving patterns through the selected intersection. These characteristics imply that the CN+ dataset has captured the realistic driving behavior of people as expected in the real world and validates the reliability of the dataset.

## Usage Notes

In this section, we provide detailed instructions to use the SUMO format of the CN+ dataset. The SUMO converted format was developed using SUMO version 1.4.0 and tested with SUMO version 1.11.0. The main SUMO Format folder (shown in Fig. 3) contains two sub-folders: ‘Infrastructure Files’ and ‘Scenario Files’. There are three XML files included in the ‘Infrastructure File’ folder. The file ‘CN+_Dataset.add.xml’ contains information about the traffic lights, bus-stops and tram-stops. All road network based information is included in the ‘CN+_Dataset.net.xml’ file. Finally, ‘CN+_Dataset.sumocfg’ file contains SUMO configuration details, and this file can be directly uploaded to the SUMO simulator.

The vehicle mobility information from the CN+ dataset is included in the ‘Scenario Files’ folder. We converted all selected one-hour-long experiments using a Python script to SUMO accepted ‘rou.xml’ files as shown in Fig. 3. All these files are named according to the date and time information inside CN+ dataset. Users can select which scenario to run in the SUMO simulator out of these 49 scenarios. Open the ‘CN+_Dataset.sumocfg’ file and add the correct path for the files ‘CN+_Dataset.add.xml’ and ‘CN+_Dataset.net.xml’. Next, add the path and the file name of the selected scenario from the ‘Scenario File’ folder to the ‘CN+_Dataset.sumocfg’ file. Once the ‘CN+_Dataset.sumocfg’ file is correctly configured, the user can open and run ‘CN+_Dataset.sumocfg’ file directly from the SUMO GUI.

Moreover, by using the CN+ dataset with simulations, accurate results can be achieved due to the realistic driving characteristics of the people. To the best of our knowledge, the CN+ dataset is one of the significant intersection-based datasets available in the VANETs domain. The CN+ dataset can be especially used in applications that focus on optimising the channel load in varying traffic conditions to meet the expected latencies, traffic management applications to control traffic congestion algorithms, applications focusing on efficient vehicular safety message broadcasting for DENM (Decentralized Environmental Notification Message), CAM (Cooperative Awareness Message) and CPM (Collective Perception Message) and many other VANET related applications including applications that analyse the effect of vehicles on the environment (C0_2_ emissions, fuel consumption, waiting times, etc.). Therefore, the CN+ dataset can be a valuable resource to many researchers in the community to simulate VANET scenarios.

## Data Availability

The codes used for this study are available on GitHub https://github.com/ThenukaRamesh/CN-_Dataset_SUMO_Conversion.
